# Optimization of Cancer Risk Assessment Models for PM_2.5_-Bound PAHs: Application in Jingzhong, Shanxi, China

**DOI:** 10.3390/toxics10120761

**Published:** 2022-12-07

**Authors:** Hongxue Qi, Ying Liu, Lihong Li, Bingqing Zhao

**Affiliations:** 1 Department of Chemistry and Chemical Engineering, Jinzhong University, Jinzhong 030619, China; 2 Department of Sciences, Northeastern University, Shenyang 110819, China

**Keywords:** PM_2.5_, PAHs, cancer risk assessment

## Abstract

The accurate evaluation of the carcinogenic risk of PM_2.5_-bound polycyclic aromatic hydrocarbons (PAHs) is crucial because of the teratogenic, carcinogenic, and mutagenic effects of PAHs. The best model out of six models was selected across three highly used categories in recent years, including the USEPA-recommended inhalation risk (Model I), inhalation carcinogen unit risk (Models IIA–IID), and three exposure pathways (inhalation, dermal, and oral) (Model III). Model I was found to be superior to the other models, and its predicted risk values were in accordance with the thresholds of PM_2.5_ and benzo[a]pyrene in ambient-air-quality standards. Models IIA and III overestimated the risk of cancer compared to the actual cancer incidence in the local population. Model IID can replace Models IIB and IIC as these models exhibited no statistically significant differences between each other. Furthermore, the exposure parameters were optimized for Model I and significant differences were observed with respect to country and age. However, the gender difference was not statistically significant. In conclusion, Model I is recommended as the more suitable model, but in assessing cancer risk in the future, the exposure parameters must be appropriate for each country.

## 1. Introduction

Atmospheric fine particulate matter (PM_2.5_) is an important cause of hazy weather, and ranks as the fifth-leading cause of global population death after hypertension, smoking, diabetes, and high total cholesterol [1]. It can also significantly increase the incidence and mortality of bronchitis, asthma, lung cancer, and other respiratory diseases [2,3]. PM_2.5_ may be attached to a variety of pollutants, such as heavy metals, sulfides, nitrogen oxides, polycyclic aromatic hydrocarbons (PAHs), and their derivatives. These toxic substances can remain deep within the human respiratory system for a long period and destroy alveolar macrophages and the immunity of the respiratory organs.

PAHs are widely present in environmental media and have been extensively studied, including their pollution characteristics, chemical composition, source analysis, health risks, and economic benefits [4]. PAHs mainly result from the incomplete combustion of mineral fuels such as coal, natural gas, and oil, with the exception a small portion of natural source emissions [5]. Owing to the teratogenic, carcinogenic, and mutagenic effects of PAHs [6], it is extremely important to assess their health risks, particularly, cancer. 

Human cancer risk assessment involves various media such as water bodies [7], soil [8], and the atmosphere [9]. The pollution of PAHs in PM_2.5_ is widespread worldwide. In some areas, such as North China [10], Brno in the Czech Republic [11], Islamabad in Pakistan [12], and Shahrrey, in Tehran, Iran, the pollution level is relatively high, and there is a high risk of cancer [13]. Accurately evaluating the carcinogenic risk of PM_2.5_-bound PAHs is crucial, and six models of three types have been frequently used in recent years. Model I, recommended by the US EPA, assesses cancer risk [14]. Only inhalation exposure is used to estimate the carcinogenic risk of pollutants [15]. Model II assesses the inhalation (carcinogenic) unit risk, and can be divided into four types according to the different inhalation unit risks of benzo[a]pyrene (BaP) (IUR_BaP_) values: IIA (8.7 × 10^−5^) [16], IIB (1.1 × 10^−6^) [17], IIC (8.0 × 10^−7^) [18], and IID (6.0 × 10^−7^) [19]. Model III is a comprehensive assessment of the carcinogenic risk of pollutants through the cumulative addition of three exposure pathways (inhalation, dermal, and oral intake) [10]. However, several questions remain, including regarding the consistence of the calculated risk values using the models, as there may be considerable differences between them [20]. The hypothesis is that only one model is best for risk assessment. However, the most representative model is yet to determined, warranting further research to accurately assess cancer risk.

Jinzhong City, located in Shanxi Province, China, has experienced frequent heavy air pollution over the past decade, particularly during winter and spring. PM_2.5_ is the predominant pollutant in this area accounting for 78.3% of the total pollution during 2016–2018 among six routinely monitored pollutants (PM_2.5_, PM_10_, SO_2_, NO_2_, CO, and O_3_) [18]. Coal combustion and secondary particles are the dominant sources of pollution. Therefore, PM_2.5_ samples were collected to measure the concentrations of PAHs using Jinzhong City as the study area, and the cancer risk values of each model were calculated according to the toxic equivalent concentration of benzo[a]pyrene (BaP) (BaP_eq_). The aim was to select the best model by analyzing the statistical differences in cancer risk values among them. Simultaneously, the population exposure parameters of the best model were optimized to provide scientific guidance for accurately assessing the cancer risk of PM_2.5_-bound PAHs in the future.

## 2. Methods

### 2.1. Sample Collection

PM_2.5_ samples were collected using a medium flow sampler (KY-2030, Qingdao Kaiyue Environmental Protection Equipment Co., Ltd., Qingdao, China), their concentrations were calculated using the gravimetric method, and the PAH content in PM_2.5_ was measured by gas chromatography–mass spectrometry (GC–MS). The detailed information is described as follows: The sampling site was located on the roof of Jinzhong University (Jinzhong City, China), approximately 15 m above the ground, and was surrounded by pollution-free emission sources. A total of 39 PM_2.5_ samples were collected in the winter of 2020, and each sample was continuously sampled for 24 h using a quartz fiber film with a sampling flow rate of 100 L·min^−1^. After sampling, the film was wrapped in tin foil and maintained at a constant temperature and humidity for 24 h. The 24 h average concentrations of PM_2.5_ were calculated using gravimetric measurement, where the sensitivity of the analytical balance was 0.01 mg, and then stored at −20 °C until chemical analysis within 2 months. 

### 2.2. Extraction and Purification

The classic Soxhlet extraction method was used to extract organic matter from the PM_2.5_. First, 80 mL of dichloromethane and *n*-hexane (1:9, *v*/*v*) (chromatography grade, Fisher Scientific (USA)) were added, and the mixture was extracted by circulation reflux for eight cycles. After natural cooling, the mixture was concentrated to 6 mL and then filtered with anhydrous sodium sulfate to concentrate it to approximately 1 mL, which was then purified. Samples were purified using a neutral silica gel chromatography column filled with dichloromethane. After sample loading, the target compounds were eluted using 30 mL of a mixture of dichloromethane and *n*-hexane (2:3, *v*/*v*). The eluents were concentrated by nitrogen blowing and *n*-hexane was added as a new solvent. The capacity of each sample was determined to be 0.5 mL for analysis. 

### 2.3. Quantitative Analysis of PAHs

PAH concentrations were quantitatively analyzed using GC–MS with electron ionization and selected ion monitoring (SCION 456-GC, Bruck Technology Co., Ltd.), according to China’s recommended standard for the determination of atmospheric PAHs using GC–MS [21]. The chromatography column was a DB-5MS (30 m × 0.25 mm, 0.25 μm), the carrier gas was argon, and the flow rate was 1 mL·min^−1^. Splitless pulse injection was performed with a pulse pressure of 250 kPa, injection temperature of 280 °C, and detector temperature of 250 °C. The internal standard method was used to calculate PAHs. The internal standards used were naphthalene-D_8_, acenaphthene-D_10_, phenylene-D_10_, chrysene-D_12_, and perylene-D_12_. Standards for the 16 PAHs were purchased from Beijing Manhage Biotechnology Co., Ltd. (Beijing, China)

### 2.4. Quality Control and Quality Assurance

All the glass instruments, quartz fiber membranes, and anhydrous sodium sulphate were baked in a muffle oven at 450 °C for 4 h before use to ensure that no external impurities were introduced during the experiment. The solvent blank (Q1), blank labelling, and repetition (Q2–Q4) were used to evaluate the pollution factors and accuracy of the experiment. The recovery rate was calculated by quantifying the percentage of the recovery indicators (2-fluorobenzene, *p*-tribiphenyl-D_14_, and benzo[a,h]pyrene-D_12_) relative to the pre-added contents after treatment in the blank sample. No other compounds were detected in the blank samples (Q1), except for the internal standard. The percentages of recovery indicators and standard samples ranged from 85.1% to 100.2% for blank labelling and repetition (Q2–Q4).

The curves of the standard PAH samples (50–1000 ng·mL^−1^) showed good linearity, and all correlation coefficients were higher than 0.999. In the instrument measurement process, a standard product was measured every 10 samples, and its relative difference with the calibration curve was within 20%, in line with the quality control requirements.

### 2.5. Cancer Risk Assessment

The incremental lifetime cancer risk (ILCR) was used to evaluate the health risk of the PAHs, and the calculation formulas of the three models were as follows: 

Model I [22]:

ILCR = (BaP_eq_ × CSF × IR × ET × EF × ED × CF)/(BW × AT) (1)

Model II [16]:

ILCR = BaP_eq_ × IUR_BaP_ (2)

Model III [10]:

ILCR_(inh)_ = [BaP_eq_ × CSF_(inh)_ × (BW/70)^1/3^ × IR × EF × ED]/(BW × AT×PEF) (3)

ILCR_(dem)_ = [BaP_eq_ × CSF_(dem)_ × (BW/70)^1/3^ × SA × AF × ABS × ED × EF]/(BW × AT × 10^6^) (4)

ILCR_(ing)_ = [BaP_eq_ × CSF_(ing)_ × (BW/70)^1/3^ × IR_(soil)_ × EF × ED]/(BW × AT × 10^6^) (5)

ILCR_(sum)_ = ILCR_(inh)_ + ILCR_(dem)_ + ILCR_(ing)_ (6)

BaP_eq_ in Equations (1)–(6) is the total BaP toxic equivalency concentration of PAH concentration in the sample, which is calculated using Equation (7):

(7) $${\mathrm{BaP}}_{\mathrm{eq}}=\sum_{i=1}^{n}C_{i}\cdot {\mathrm{TEF}}_{i}$$

In Formula (7), *C_i_* is the concentration of each PAH compound in the sample. The units of *C_i_* are ng·m^−3^ in Models I and II and mg·kg^−1^ in Model III. TEF*_i_* is the BaP toxic equivalent factor (TEF) of each PAH compound in the samples [23].

The other variables, units, and their values in Equations (1)–(6) are listed in Table 1. According to the US EPA regulations [22], an ILCR < 10^−6^ is considered an acceptable level (very low risk), with other definitions as follows: low risk (10^−6^ ≤ ILCR < 10^−4^), moderate risk (10^−4^ ≤ ILCR < 10^−3^), high risk (10^−3^ ≤ ILCR < 10^−1^), and very high risk (ILCR ≥ 10^−1^).

### 2.6. Statistical Analysis

Data of the PM_2.5_ and PAH concentrations were reported as the mean ± standard deviation. The incremental lifetime cancer risk (ILCR) values of the individual groups were in line with a normal distribution and homogeneity of variance after a common logarithmic transformation. Significant differences in the risk values among the models were analyzed using one-way analysis of variance, and multiple comparisons were performed using Tukey’s test with SPSS 26.0 software.

## 3. Results and Discussion

### 3.1. Levels of PM_2.5_ and Their Bound PAHs

The daily concentrations of PM_2.5_ are presented in Table S1 and their statistical description is presented in Table 2, they ranged from 21.4 to 183 μg·m^−3^ in 39 samples, with a median of 61.1 μg·m^−3^ and a mean of 70.2 ± 33.1 μg·m^−3^. All samples exceeded Grade I (35 μg·m^−3^) of the Chinese ambient-air-quality standard [21], and 64% of the samples exceeded the Grade II (75 μg·m^−3^) [21] and interim Grade I standards (75 μg·m^−3^) of the *WHO global air quality guidelines* [24]. These results indicate that air pollution during winter was severe in this area. 

The daily contents of 16 PAHs and BaP_eq_ are also presented in Table S1 and their statistical description is presented in Table 2. Concentrations of the 16 PAHs ranged from 1.92 to 183 ng·m^−3^, with a median of 38.4 ng·m^−3^ and an average concentration of 48.8 ± 40.4 ng·m^−3^. Accordingly, BaP_eq_ ranged from 0.16 to 11.1 ng·m^−3^, with a median of 2.59 ng·m^−3^ and a mean of 2.85 ± 2.62 ng·m^−3^. The overall level was lower than in previous reports, in which the BaP_eq_ values ranged from 0.79 to 139.8 ng·m^−3^, with an average of 17 ng·m^−3^ in 67 cities in China from 2001 to 2016 [4]. Higher BaP_eq_ values have been reported for Jinzhong City.

The composition of PAHs in PM_2.5_ is affected by the local energy structure, economic development, and atmospheric conditions [25]. Therefore, the data had different characteristics such as spatial distribution [26] and seasonal variations [27]. For instance, Yan et al. (2019) found that in 67 cities and regions in China, the contents of PM_2.5_-bound PAHs in northern cities were lower than those in southern cities, and they were higher in winter than in summer in terms of geographic and temporal distribution [4]. In the present study, among the 16 PAHs, the average concentration of pyrene (7.56 ng·m^−3^) was the highest, accounting for 15.37%. This was followed by anthracene, accounting for 14.19%. The order of the proportion of each ring number was as follows: 4-ring (41 ± 21%) > 3-ring (19 ± 12%) > 2-ring (15 ± 18%) > 6-ring (15 ± 14%) > 5-ring (12 ± 7%). The 2-ring and 3-ring PAHs accounted for a relatively low proportion owing to their low molar mass, high volatility, strong migration ability, and difficulty of adhesion. However, 4-ring PAHs were less volatile than 2-ring and 3-ring PAHs, and the increase in coal burning sources in the winter resulted in the increase of 4-ring PAHs, which accounted for the largest proportion.

### 3.2. Selection of Models of Cancer Risk Assessment

#### 3.2.1. Statistical Differences among the Models of Cancer Risk Assessment

**Figure 1:**
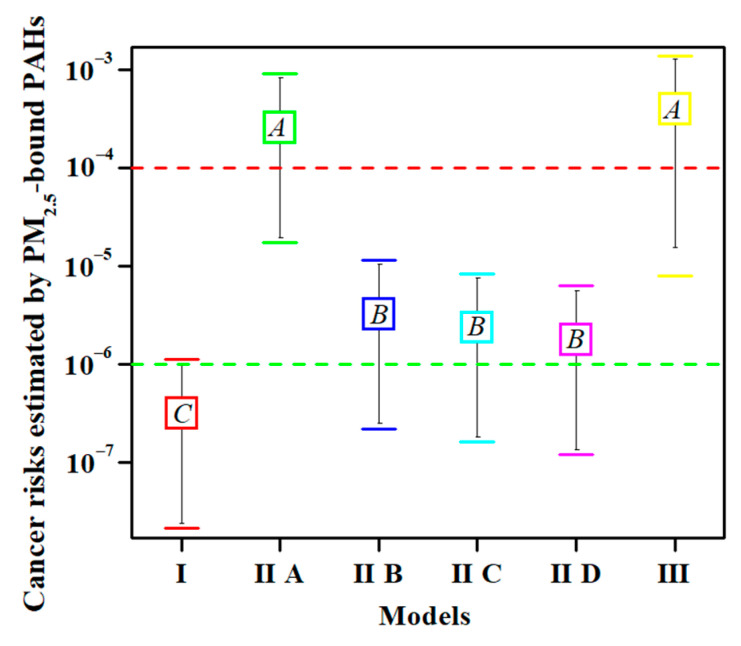
The analysis of variance and multiple comparisons among the total cancer risks calculated using the different models (*n* = 39, *p* < 0.01). Note: The upper and lower horizontal lines in each group represent upper and lower limits, respectively. The box represents the average value. The same letter in the box indicates that the difference is not significant. One-way analysis of variance was used to determine the significance of differences, and Tukey’s test was used for multiple comparisons. The green dotted line represents an acceptable level of cancer risk. The red dotted line represents moderate risk.

The carcinogenic risk values for each model were calculated using the same concentrations of 16 PAHs in the 39 samples. The units of PAH content are ng·m^−3^ in Models I and II (Table S1) and mg·kg^−1^ in Model III (Table S2). As shown in Figure 1, the six models were divided into three groups according to multiple comparisons and labelled as *A*, *B*, and *C*. No significant difference was observed within the groups (*n* = 39, *p* < 0.01); for example, those between Models IIA and III. Similarly, no significant differences were found among Models IIB, IIC, and IID. However, the differences between groups reached highly significant levels (*n* = 39, *p* < 0.01), such as between Models I and IIA, and between Models I and IIB. These results suggest that some of the cancer risks calculated using the above models were different and could not be substituted for each other. Further research is required to determine the optimal model.

#### 3.2.2. Advantages of Model I

The risk values of Model I were consistent with the thresholds of PM_2.5_ and BaP. In detail, the 24 h average concentrations of PM_2.5_ in 64% of the samples were higher than the Grade II Chinese air quality standards (75 μg·m^−3^) [29]. In addition, 72% of the BaP_eq_ concentrations exceeded the concentration limit of BaP set by the WHO (1 ng·m^−3^) [30], while 48% of the samples exceeded the Chinese air quality standard of 2.5 ng·m^−3^ [29]. Correspondingly, the risk values of 5% of the samples in Model I were greater than 10^−6^, indicating a certain potential risk. Overall, the results of the above three aspects (PM_2.5_, BaP_eq_, and cancer risk) were consistent and mutually supportive without contradiction. Model I has been used for cancer risk assessment in Anshan [31], Beijing [32], Caofeidian [33], Shijiazhuang [34], and Wuhan [35]. Model I provided a good risk warning in these areas and should be the optimal model for cancer risk assessment for PM_2.5_-bound PAHs.

#### 3.2.3. Simple and Convenient Models IIB, IIC, and IID

The primary advantage of Models IIB, IIC, and IID is the convenience of calculation, as only the IUR_BaP_ value is multiplied by the BaP_eq_ value. However, a disadvantage of this study is that age and sex differences are excluded. Although each of the three models was independently used to assess the carcinogenic risk of PAHs in PM_2.5_ in some areas, there was no statistically significant difference in the risk values among models IIB, IIC, and IID (Figure 1). This result suggests that only one of the three models can completely replace the other two. The question arises as to which model is best for use in the actual calculations. As previously reported, an IUR_BaP_ value of 1.1 × 10^−6^ (Model IIB) was used to assess cancer risk in Beijing, China [36], Heilongjiang, China [37], Czech Republic [11], Monterrey, Mexico [38], Northern Thailand [20], and Shiraz, Iran [39]. This model was obtained from the Office of Environmental Health Hazard Assessment of the California Department of Environmental Protection [17]. An IUR_BaP_ value of 6.0 × 10^−7^ (Model IID) was used to assess cancer risk in Tianjin, China [40], Taiyuan, China [41], and Uttar Pradesh, India [42]. This model came from the comprehensive risk information system of the US EPA [19]. The mean of the above two IUR_BaP_ values from Models IIB and IID was 8.0 × 10^−7^ (Model IIC) [18], and was used to assess cancer risk in China [18] and Tehran, Iran [13]. As Model IIC is the average of Models IIB and IID, it can be ruled out. For Models IIB or IID, because Model IIB is from California, while Model IID is from US EPA, it is advisable to choose Model IID in future risk assessment. In summary, Model IID should be used in the calculation of inhalation carcinogenic risk because it can replace the results of Models IIB and IIC.

#### 3.2.4. Model IIA and Model III Overestimated Cancer Risk

Although the estimated cancer risks should not be equivalent to the actual cancer incidence, they have important reference values and positive significance for the probability of individual cancers in the population. The local incidence of cancer was obtained from the Yuci District, Jinzhong City, Shanxi Province, China. The actual incidences of cancer were 222.26 per 100,000 people in 2011 [43], 245.03 in 2012 [43], 240.29 in 2013 [43], 282.15 in 2014 [44], 281.33 in 2015 [45], and 216.27 in 2016 [46]. In other words, the rates of local cancer incidence were in the range of (2.16–2.82) × 10^−3^. The etiology of cancer is complex and includes behavioral, dietary, metabolic, environmental, infectious, and other factors [47]. As previously reported, the main risk factors for cancer in China are chronic infection, smoking, low fruit and vegetable intake, alcohol consumption, and occupational exposure. The remaining factors, including environmental agents, physical inactivity, use of exogenous hormones, and reproductive factors, accounted for less than 1.0% each [48]. According to the above results, the risk of chemical contamination was less than 1%. If estimated at a maximum value of 1%, the maximum risks of the environmental agents were approximately 2.16–2.82 × 10^−5^. However, the average cancer risks for adults were 2.57 ± 2.33 ×10^−4^ in Model IIA and 3.92 ± 3.98 × 10^−4^ in Model III, representing moderate risk [22]. The risk values calculated by Models IIA and III were one order of magnitude higher than the risks of environmental agents estimated by the actual local incidences, suggesting that Models IIA and III might overestimate cancer risks. Chemical carcinogens in the environment are not only PAHs, but also heavy metals and other organic pollutants [28].

Moreover, the concentration limit of BaP regulated by the WHO is 1 ng·m^−3^ [30], and the threshold in the Chinese air quality standard is 2.5 ng·m^−3^ [29]. The mean value of BaP_eq_ in the present study was 2.85 ± 2.62 ng·m^−3^, which was similar to the thresholds of the above two criteria. However, higher risk values (approximately 10^−4^) could be calculated using Models IIA and III. Similar results have been reported in previous studies (Table 3), in which the average BaP content was in the range of 1–10 ng·m^−3^, and carcinogenic risk values of approximately 10^−4^ were calculated using Model IIA in Brno, Czech Republic [11], Islamabad, Pakistan [12], Shahrrey, Iran [13], Nan Province, Thailand [20], and Wuhan, China [49]. Analogous results were obtained for Model III in North China [10], Huanggang, China [50], and Jinzhong, China.

A previous study also reported that Model IIA might have overestimated the cancer risk of nonoccupational exposure, because it used epidemiological data on occupational exposure of coke-oven workers [37] and the risks are considerable higher in occupationally exposed people than in the general population. This could explain why Model IIA overestimated the risk of cancer, and the parameters of nonoccupational exposure should be re-optimized in the future to accurately assess cancer risk.

In Model III, despite oral and dermal exposure being considered in addition to inhalation, the cancer risk of exposure to PM_2.5_ mainly resulted from dermal exposure and ingestion instead of inhalation. For instance, the average contribution of inhalation exposure in adults was less than 1% of the total cancer risk in the present study. Similar results were also reported in previous studies, and the cancer risk values of dermal exposure were two orders of magnitude [51,52] and approximately 10^3^–10^4^ times [10,53] greater than those of inhalation exposure. However, inhalation is the predominant pathway for PM_2.5_ [54]. Another difference was that the unit of BaP_eq_ was mg·kg^−1^ in Model III instead of ng·m^−3^. Therefore, Model III overestimates the cancer risk for atmospheric PM_2.5_, and is more suitable for cancer risk assessment of pollutants in soil or dust particles, because oral and dermal exposures represent the predominant pathways instead of inhalation exposure in Model III.

Overall, based on statistical analysis, comparison with actual cancer incidence, and air quality standards, Model I was considered the best model among the models. Therefore, the next step was to optimize the exposure parameters of Model I.

### 3.3. Selection of Population Exposure Parameters in Model I

The optimization of exposure parameters in Model I was performed according to the presence of significant differences among countries, sexes, and ages. The population exposure parameters for different countries, including the USA [22], Australia [55], Japan [56], Korea [57], and China [58,59], were collected and are summarized in Table 4.

As shown in Table 5, from multiple comparisons of the significant differences among countries, the total ILCR values of adults in both the USA and China were significantly higher than those in Japan and Korea, whereas those in Australia were at a median level. However, regarding sex, no significant differences were observed between women and men in the USA, Korea, or China. Moreover, there were no significant differences between women and adults, or between men and adults.

As shown in Table 6, age did not exhibit significant differences between adults in the USA and China; however, the differences were significant among children. Furthermore, significant differences were found between adults and children in the USA, with average values of 3.15 ± 2.86 × 10^−7^ and 8.48 ± 7.69 × 10^−8^ for adults and children, respectively. Similar results were observed in China, with significant differences between adults and children of all ages; however, there was no significant difference within the age groups in China. This is attributed to the inhalation rate and body weight being related in the calculation formula of Model I, despite both factors increasing with age [22]. Thus, it had little influence on the changes in total average daily dose (ADD) values. For instance, the corresponding ADD values for children of ages 6–9, 9–12, 12–15, and 15–18 in China were 0.66, 0.96, 0.98, and 1.03, respectively. The difference was in the range of 1.6.

These results indicate that the population exposure parameters recommended by the US EPA cannot be directly used in cancer risk assessments. Therefore, the parameters for each country were used. Some parameters vary significantly among countries. For example, the exposure time of Americans was 4 h [22], while that of China was 3.68 h [59]. However, Japanese and Korean people spend less time outdoors, with exposure times of 1.2 h [56] and 1.3 h [57], respectively. The difference between the two was three-fold. In addition, age differences are not negligible, and cancer risk should be assessed separately for adults and children. However, the sex difference was not significant; females and males do not need to be treated separately, and the cancer risk of adults should be assessed in the future.

### 3.4. Limitations

The composition of PM_2.5_ is very complex, and organic pollutants such as PAHs are only a small part of PM_2.5_, which also includes ammonium, chlorine, elemental carbon, heavy metals, nitrate, and sulfate [60]. In the present study, only exposure to PAHs was assessed for carcinogenic risk, although the health effects of other compounds should not be ignored. Furthermore, the additive effect was used to evaluate the carcinogenic risk of 16 PAHs. However, the synergistic or antagonistic effects may also occur during metabolic processes [61,62]. The above deficiencies should be overcome to assess the effects of PAHs on human health accurately in future.

## 4. Conclusions

Using the same group of PAH data, the carcinogenic risk values of PAHs in PM_2.5_ were calculated using six models in three categories frequently used in recent years. They were analyzed for statistical significance and compared with actual cancer incidence in the local population. Model I was superior to the other models, and its predicted risk values were in accordance with the limits of PM_2.5_ and BaP in ambient-air-quality standards. Models IIA and III overestimated the risk of cancer, and their parameters should be optimized again to accurately assess cancer risk. Model IID of the USEPA’s integrated risk-information system can replace the results of Models IIB and IIC because there is no statistically significant difference among Models IIB, IIC, and IID. In addition, the population exposure parameters recommended by the USEPA cannot be used directly, and the parameters were optimized for Model I. The country and age differences must be considered in future risk assessment. However, gender differences can be ignored because no significant differences were found between the sexes. Overall, Model I is recommended as the best model, but assessing cancer risk in the future, the exposure parameters must be appropriate for each country. The correct selection of the cancer risk assessment model for PM_2.5_-bound PAHs can improve the accuracy of health risk assessment and provide a scientific reference for emergency strategies for heavy pollution weather in winter.

## Figures and Tables

**Table 1:** Exposure parameters and their values in models of assessed carcinogenic risk.

Model	Parameter	Abbreviation	Unit	Adult
I [22]	Carcinogenic slope factor	CSF	[mg/(kg·d)] ^−1^	3.14
	Inhalation rate	IR	m^3^·d^−1^	20
	Exposure time	ET	h·d^−1^	4
	Exposure frequency	EF	d·a^−1^	350
	Exposure duration	ED	a	52
	Conversion factor	CF	—	10^−6^
	Body weight	BW	kg	70
	Averaging time	AT	a	70
IIA [16]	Inhalation unit risk	IUR_BaP_	(ng·m^−3^)^−1^	8.7 × 10^−5^
IIB [17]				1.1 × 10^−6^
IIC [18]				8.0 × 10^−7^
IID [19]				6.0 × 10^−7^
III [10]	Carcinogenic slope factor (inhalation)	CSF_(inh)_	[mg/(kg·d)] ^−1^	3.85
	Exposure duration	ED	a	24
	Particulate emission factor	PEF	m^3^·kg^−1^	1.36 × 10^9^
	Exposed skin area	SA	cm^2^	5800
	Skin attachment factor	AF	mg·cm^−2^·d	0.07
	Skin adherence factor	ABS	—	0.13
	Carcinogenic slope factor	CSF_(dem)_	[mg/(kg·d)] ^−1^	25
	Ingestion rate	IR_(soil)_	mg·d^−1^	100
	Cancer slope factor (ingestion)	CSF_(ing)_	[mg/(kg·d)] ^−1^	7.3

—: Dimensionless.

**Table 2:** Statistical description of polycyclic aromatic hydrocarbon (PAH) concentrations in PM_2.5_ and toxic equivalency concentrations of benzo[a]pyrene (BaP_eq_) (ng·m^−3^, *n* = 39).

		Ring	Class ^a^	TEF ^b^	Detection Rate/%	Min	Median	Max	Mean	SD
1	Naphthalene	2	2B	0.001	97	0.13 ^c^	3.25	70.7	6.91	12.2
2	Acenaphthylene	3	— ^d^	0.001	82	0.04	0.72	16.1	1.58	3.02
3	Fluorene	3	3	0.001	62	0.07	0.34	1.51	0.48	0.38
4	Acenaphthene	3	3	0.001	74	0.06	1.63	14.4	2.98	3.56
5	Phenanthrene	3	3	0.001	59	0.06	0.71	6.13	1.56	1.71
6	Anthracene	3	3	0.01	85	0.09	3.81	19.5	6.28	6.17
7	Fluoranthene	4	3	0.001	97	0.09	3.99	21.4	6.11	6.33
8	Pyrene	4	3	0.001	100	0.06	4.33	20.7	5.83	6.14
9	Benz[a]anthracene	4	2B	0.1	100	0.12	0.42	5.48	0.98	1.25
10	Chrysene	4	2B	0.01	77	0.07	4.48	21.8	6.02	5.90
11	Benzo[b]fluoranthene	5	2B	0.1	77	0.08	2.89	24.7	4.84	5.92
12	Benzo[k]fluoranthene	5	2B	0.1	74	0.05	1.72	10.3	2.41	2.33
13	Benzo[a]pyrene	5	1	1.0	85	0.06	0.39	2.19	0.53	0.52
14	Dibenzo[a,h]anthracene	5	2A	1.0	100	0.06	0.68	4.73	1.13	1.23
15	Indeno[1,2,3-c,d]pyrene	6	2B	0.1	100	0.08	3.06	33.4	6.06	7.78
16	Benzo[g,h,i]perylene	6	3	0.01	64	0.07	1.00	5.32	1.65	1.48
	∑PAH					1.92	38.4	154	48.1	38.0
	BaP_eq_ (ng·m^−3^)					0.16	2.59	11.1	2.85	2.62
	BaP_eq_ (mg·kg^−1^)					0.74	20.4	138	31.9	34.6
	PM_2.5_ (μg·m^−3^)					21.4	61.1	183	70.2	33.1

^a^ Class 1 carcinogen: confirmed carcinogen for humans; Class 2A: evidence of carcinogenicity to humans is limited, but evidence of carcinogenicity to experimental animals is sufficient; Class 2B: evidence of carcinogenicity to humans is limited, evidence of carcinogenicity to experimental animals is insufficient; and Class 3: suspected carcinogenicity to humans, with insufficient human or animal data [28]. ^b^ TEF: toxic equivalence factor; literature from Nisbet and Lagoy 1992 [23]. ^c^ The detection limit was 0.035 ng·m^−3^. ^d^ —: Not reported.

**Table 3:** Mean concentrations (± standard deviation) or range (min–max) of BaP_eq_ and cancer risks for adult from the inhalation exposure of PM_2.5_-bound 16 PAHs compared to other countries and regions.

Model	Country	Area	Year	Samples	BaP_eq_ (ng·m^−3^)	ILCR (×10^−4^)	Reference
	Czech	Brno	2017	15	3.47 (0.495–11.7)	3.02 (0.43–10.16)	[11]
	Pakistan	Islamabad	2017	160	4.45	6.39	[12]
	Iran	Shahrrey, Tehran	2019	45	7.09 ± 5.82	6.17	[13]
IIA	Thailand	Nan Province	2018	40	1.54 ± 1.36	1.34	[20]
	China	Wuhan	2015	115	3.48 ± 3.53	3.03	[49]
	China	Jinzhong, Shanxi	2021	39	2.96 ± 2.68	2.57 ± 2.33	This study
	China	North China	2015	90	2.94	1.99	[10]
III	China	Huanggang	2018	28	4.08 ± 2.22	0.37 ± 0.20	[50]
	China	Jinzhong, Shanxi	2021	39	2.85 ± 2.62	3.92 ± 3.98	This study

**Table 4:** Exposure parameters and their values for carcinogenic risk assessment in Model I.

		CSF	IR	ET	EF	ED	BW	AT	CF	ADD (×10^−8^)
	Adults	3.14	20	4	350	52	70	70	10^−6^	10.7
USEPA	Females	3.14	14.7	4	350	52	73	80.4	10^−6^	6.54
[22]	Males	3.14	14.7	4	350	52	86	75.4	10^−6^	5.92
	Children	3.14	10	4	350	6	15	70	10^−6^	2.87
Australia [55]	Adults	3.14	16	3	350	52	75	81.5	10^−6^	5.12
Japan [56]	Adults	3.14	17.3	1.2	350	52	58.4	81.2	10^−6^	2.86
Korea	Adults	3.14	14.3	1.3	350	52	62.8	78.6	10^−6^	2.46
[57]	Females	3.14	12.8	1.3	350	52	56.4	81.9	10^−6^	2.35
	Males	3.14	15.7	1.3	350	52	69.2	75.1	10^−6^	2.56
	Adults	3.14	15.7	3.68	350	52	60.6	74.8	10^−6^	8.32
China	Females	3.14	14.5	3.48	350	52	56.8	77.4	10^−6^	7.49
[58,59]	Males	3.14	18	3.93	350	52	65	72.4	10^−6^	9.81
	Children (6–9)	3.14	10.1	1.73	350	6	26.5	74.8	10^−6^	0.66
	Children (9–12)	3.14	13.2	1.77	350	9	36.8	74.8	10^−6^	0.96
	Children (12–15)	3.14	13.5	1.7	350	12	47.3	74.8	10^−6^	0.98
	Children (15–18)	3.14	14	1.3	350	15	54.8	74.8	10^−6^	1.03

CSF: carcinogenic slope factor, [mg/(kg·d)]^−1^; IR: inhalation rate, m^3^·d^−1^; ET: exposure time, h·d^−1^; EF: exposure frequency, d·a^−1^; ED: exposure duration, a; BW: body weight, kg; AT: averaging time, a; CF: conversion factor, ADD: average daily dose, m^3^·ng^−1^.

**Table 5:** The analysis of variance and multiple comparisons among the total cancer risks calculated using Model I based on the different countries (*n* = 39).

Country	Population	Mean ± SD	*p* < 0.05
	Adults	(3.15 ± 2.86) × 10^−7^	*A*
USA	Females	(1.93 ± 1.75) × 10^−7^	*AB*
	Males	(1.75 ± 1.59) × 10^−7^	*AB*
Australia	Adults	(1.51 ± 1.37) × 10^−7^	*AB*
Japan	Adults	(8.44 ± 7.66) × 10^−8^	*B*
	Adults	(7.26 ± 6.59) × 10^−8^	*B*
Korea	Females	(6.95 ± 6.30) × 10^−8^	*B*
	Males	(7.57 ± 6.87) × 10^−8^	*B*
	Adults	(2.46 ± 2.23) × 10^−7^	*A*
China	Females	(2.21 ± 2.01) × 10^−7^	*A*
	Males	(2.90 ± 2.63) × 10^−7^	*A*

Data from each group were normally distributed and homogenous in variance after being converted to log values. One-way analysis of variance was used to determine the significance of differences, and Tukey’s test was used for multiple comparisons. The same letter in the same column indicates that the difference was not significant.

**Table 6:** The analysis of variance and multiple comparisons among the total cancer risks calculated using Model I based on age (*n* = 39).

Country	Population	Mean ± SD	*p* < 0.01
USA	Adults	(3.15 ± 2.86) × 10^−7^	*A* ^a^
	Children	(8.48 ± 7.69) × 10^−8^	*B*
	Adults	(2.46 ± 2.23) × 10^−7^	*A*
China	Children (6–9)	(1.96 ± 1.78) × 10^−8^	*C*
	Children (9–12)	(2.83 ± 2.57) × 10^−8^	*C*
	Children (12–15)	(2.89 ± 2.62) × 10^−8^	*C*
	Children (15–18)	(3.04 ± 2.76) × 10^−8^	*C*

^a^ Data from each group were normally distributed and homogenous in variance after being converted to log values. One-way analysis of variance was used to determine the significance of differences, and Tukey’s test was used for multiple comparisons. The same letter in the same column indicates that the difference was not significant.

## Data Availability

Not applicable.

## Supplementary Materials

The following supporting information can be downloaded at: https://www.mdpi.com/article/10.3390/toxics10120761/s1. Table S1: PM_2.5_ mass (μg·m^−3^) and its polycyclic aromatic hydrocarbon (PAH) concentrations (ng·m^−3^) during winter 2020 (*n* = 39); Table S2: PM_2.5_ mass (μg·m^−3^) and its polycyclic aromatic hydrocarbon (PAH) concentrations (mg·kg^−1^) during winter 2020 (*n* = 39).
