# Supplementary material for: Optimization of Cancer Risk Assessment Models for PM2.5-Bound PAHs: Application in Jingzhong, Shanxi, China
Source: Toxics. 2022 Dec 7;10(12):761. doi: 10.3390/toxics10120761 (PMC9781926; doi:10.3390/toxics10120761)
Supplement: Supplementary file 1 [file toxics-10-00761-s001.zip › toxics-2025425-supplementary.pdf]

Supplementary Materials: Optimization of Cancer Risk Assessment Models for $PM_{2.5}$-Bound PAHs: Application in Jingzhong, Shanxi, China

Hongxue Qi, Ying Liu [2,*], Lihong Li* and Bingqing Zhao

Table S1 $PM_{2.5}$ mass ($\mu g \cdot m^{-3}$) and its polycyclic aromatic hydrocarbons (PAHs) concentrations ($ng \cdot m^{-3}$) during winter in 2020 (*n* = 39)

	Sample No.	1	2	3	4	5	6	7	8	9	10	11	12	13	14	15	16	17	18	19	20
	Date	11.3	11.4	11.5	11.6	11.7	11.8	11.9	11.11	11.13	11.14	11.15	11.16	11.18	11.19	11.2	11.21	11.22	11.23	11.24	11.25
	$PM_{2.5}$	44	75	85	54	63	35	74	90	106	58	61	120	28	28	83	54	28	54	142	183
1	Naphthalene	0.19[a]	3.25	1.70	3.88	0.91	0.75	0.44	1.09	3.32	0.23	1.67	0.29	4.22	1.17	0.13	1.06	4.72	4.49	1.42	4.94
2	Acenaphthylene	0.23	0.76	1.62	0.19	—	—	—	—	2.82	0.05	0.05	0.08	0.04		1.90	1.56	2.53	0.48	0.09	0.77
3	Fluorene	0.10	0.76	0.09	0.07	0.78	—	—	—	0.10	—	—	—	—	0.21	0.10	0.35	0.71	—	—	0.24
4	Acenaphthene	0.85	1.96	0.17	1.90	1.96	—	—	0.10	0.28	0.19	—	—	0.22	0.46	4.51	—	0.57	—	—	1.41
5	Phenanthrene	0.17	0.06	0.66	0.53	0.33	—	—	—		—	—	—	—	—	0.20	0.12	0.32	—	—	1.39
6	Anthracene	2.10	1.70	12.37	1.39	0.74	—	—	—	0.44	0.70	0.17	0.24	0.46	—	3.91	2.18	5.56	—	—	4.71
7	Fluoranthene	2.90	3.32	0.42	4.12	5.85	—	0.09	0.31	0.09	1.20	0.21	0.11	0.76	0.42	2.31	2.64	5.57	0.23	0.28	5.36
8	Pyrene	1.91	5.26	1.60	7.11	5.23	0.67	11.4	20.4	0.66	20.7	13.8	1.63	19.9	1.90	9.19	6.44	5.53	0.35	0.29	8.08
9	Benzo[a]anthracene	0.14	0.94	1.06	1.73	0.43	0.12	0.15	0.22	0.24	0.19	0.18	0.20	0.27	0.25	4.57	0.48	2.92	0.13	0.16	0.25
10	Chrysene	0.07	0.93	0.20	1.75	0.27	—	—	—	—	—	0.07	—	—	—	0.10	2.27	14.8	0.12	—	7.04
11	Benzo[b]fluoranthene	0.15	1.72	2.50	1.03	—	—	—	—	—	1.57	—	—	—	1.30		1.98	7.24	0.14	0.40	3.40
12	Benzo[k]fluoranthene	0.05	2.21	1.85	3.80	1.21	—	—	—	—	0.45	—	—	—	0.19	1.37	0.54	0.60	—	0.11	0.07
13	Benzo[a]pyrene	—[b]	0.57	0.34	0.44	0.26	—	0.07	0.11	0.07	—	0.09	—	0.06	0.08	0.09	0.09	0.81	—	0.07	0.56
14	Dibenzo[a,h]anthracene	0.17	0.68	1.44	0.64	0.49	0.16	0.16	0.15	0.26	1.11	0.22	0.19	0.11	0.41	0.59	0.76	4.26	0.24	0.18	1.20
15	Indeno[1,2,3-c,d]pyrene	0.24	7.47	14.1	12.7	10.5	0.22	0.25	0.09	1.34	0.27	1.43	0.11	1.52	6.48	9.05	8.19	10.4	0.29	0.08	22.2
16	Benzo[g,h,i]perylene	—	0.81	2.38	0.85	—	—	—	—	—	0.51	—	—	—	0.12	0.11	0.15	2.65	—	0.07	2.03
	∑PAH	9.08	32.4	42.5	42.1	29.0	1.92	12.6	22.5	9.62	27.2	17.9	2.85	27.6	13.0	38.1	28.8	69.2	6.47	3.15	63.7
	BaP_{eq} ($ng \cdot m^{-3}$)	0.23	1.93	2.75	2.00	1.03	0.16	0.25	0.29	0.36	1.40	0.35	0.20	0.22	0.72	1.03	1.46	7.81	0.28	0.31	3.31

[a] The reporting limit for individual compounds in $PM_{2.5}$ was 0.035 $ng \cdot m^{-3}$.

[b] —: Not detected.

Continuous **Table S1** $PM_{2.5}$ mass ($\mu g \cdot m^{-3}$) and its polycyclic aromatic hydrocarbons (PAHs) concentrations ($ng \cdot m^{-3}$) during winter in 2020 (*n* = 39)

	Sample No.	21	22	23	24	25	26	27	28	29	30	31	32	33	34	35	36	37	38	39
	Date	11.26	11.27	11.28	11.29	11.3	12.1	12.3	12.4	12.5	12.6	12.7	12.1	12.11	12.13	12.14	12.15	12.16	12.17	12.18
	$PM_{2.5}$	88	106	63	67	70	118	57	59	45	111	47	75	61	21	59	44	52	60	69
1	Naphthalene	2.15 [a]	20.9	2.26	16.8	3.25	4.64	7.50	6.73	14.8	70.7	21.9	11.2	12.5	2.85	—	8.53	6.49	0.38	2.58
2	Acenaphthylene	0.19	2.48	0.57	16.1	0.73	0.91	7.58	0.71	0.35	0.51	0.36	0.85	2.64	—	—	0.99	2.09	0.33	0.13
3	Fluorene	— [b]	0.97	—	0.41	0.11	0.28	—	—	0.73	0.24	0.49	0.89	1.51	0.32	—	—	0.72	1.08	0.29
4	Acenaphthene	0.99	—	0.60	—	1.63	1.46	0.92	4.20	14.4	—	2.64	6.06	6.59	2.77	0.06	7.33	3.81	6.57	11.8
5	Phenanthrene	—	0.15	6.13	0.71	—	2.15	—	1.91	2.41	0.59	0.71	5.04	3.88	0.59	—	1.35	3.83	2.56	—
6	Anthracene	3.70	0.94	3.31	2.60	6.94	9.08	3.59	16.1	29.3	8.05	6.15	16.1	19.5	6.94	0.09	16.7	10.1	15.4	19.1
7	Fluoranthene	4.87	0.24	3.86	3.34	15.4	13.6	6.18	20.0	21.42	8.52	7.91	13.2	13.3	6.88	0.41	16.9	10.3	11.8	17.9
8	Pyrene	1.56	0.31	2.65	0.55	8.39	4.72	3.34	11.1	20.7	0.37	2.68	5.73	9.81	2.69	0.21	0.06	4.33	5.29	1.00
9	Benzo[a]anthracene	0.27	0.15	0.40	1.36	1.59	0.70	0.53	2.72	3.27	5.48	0.42	1.27	1.66	0.34	0.14	0.34	0.73	1.22	1.06
10	Chrysene	11.1	3.34	6.61	6.13	3.60	12.6	5.01	9.21	1.06	3.95	21.8	9.63	18.3	7.75	0.90	5.29	10.2	14.3	2.26
11	Benzo[b]fluoranthene	4.77	0.28	3.52	3.11	9.24	2.15	1.01	9.96	24.7	0.44	3.22	13.6	18.8	2.67	0.08	13.4	4.82	4.16	3.88
12	Benzo[k]fluoranthene	1.70	1.50	1.12	—	3.79	0.77	0.32	3.74	10.3	5.68	2.06	4.84	6.39	1.72	—	4.67	2.93	3.31	2.47
13	Benzo[a]pyrene	0.37	1.07	0.10	1.11	0.99	0.47	0.12	0.89	1.77	2.19	0.21	0.48	1.13	0.18	—	0.65	0.51	0.39	1.02
14	Dibenzo[a,h]anthracene	2.04	1.07	1.63	1.01	4.46	2.32	1.74	2.77	0.97	0.20	0.73	1.38	4.73	0.58	0.06	0.56	0.45	0.89	3.08
15	Indeno[1,2,3-c,d]pyrene	4.49	0.48	0.83	1.47	3.25	3.06	2.60	27.9	33.4	13.3	1.47	0.71	4.92	13.4	1.77	2.17	4.91	3.51	5.77
16	Benzo[g,h,i]perylene	0.18	—	0.41	0.58	0.72	1.38	—	1.00	3.90	—	0.81	3.36	5.32	1.42	—	3.56	2.05	2.79	3.99
	∑PAH	38.4	33.9	34.0	55.3	64.1	60.3	40.4	119	154	120	73.6	94.3	131	51.1	3.72	82.5	68.3	74.0	76.3
	BaP_{eq} ($ng \cdot m^{-3}$)	4.28	2.69	2.96	3.19	7.33	4.64	2.59	6.56	7.47	3.75	3.85	5.23	11.1	2.34	0.18	4.13	3.15	3.97	5.65

[a] The reporting limit for individual compounds in $PM_{2.5}$ was 0.035 $ng \cdot m^{-3}$.

[b] —: Not detected.

Table S2 $PM_{2.5}$ mass ($\mu g{\cdot}m^{-3}$) and its polycyclic aromatic hydrocarbons (PAHs) concentrations ($mg{\cdot}kg^{-1}$) during winter in 2020 (*n* = 39)

	Sample No.	1	2	3	4	5	6	7	8	9	10	11	12	13	14	15	16	17	18	19	20
	Date	11.3	11.4	11.5	11.6	11.7	11.8	11.9	11.11	11.13	11.14	11.15	11.16	11.18	11.19	11.2	11.21	11.22	11.23	11.24	11.25
	$PM_{2.5}$	44	75	85	54	63	35	74	90	106	58	61	120	28	28	83	54	28	54	142	183
1	Naphthalene	0.61[a]	21.7	10.0	18.6	3.56	10.6	2.98	6.10	15.6	1.99	13.7	3.66	76.0	21.1	0.81	20.1	83.9	41.3	6.97	41.1
2	Acenaphthylene	0.75	5.04	9.57	0.93	—	0.29	0.04	—	13.3	0.44	0.37	0.97	0.76	0.75	11.5	29.5	45.1	4.40	0.45	6.42
3	Fluorene	0.31	5.04	0.56	0.32	3.05	—	—	—	0.46	—	—	—	—	3.71	0.60	6.51	12.6	—	—	2.00
4	Acenaphthene	2.69	13.1	0.98	9.10	7.71	—	—	0.58	1.34	1.67	—	—	4.00	8.29	27.3		10.2	—	—	11.7
5	Phenanthrene	0.54	0.37	3.89	2.55	1.29	—	—	—	—	—	—	—	—	—	1.21	2.29	5.71	—	—	11.6
6	Anthracene	6.68	11.3	73.1	6.66	2.92	2.14			2.05	6.10	1.36	2.94	8.29	—	23.7	41.1	98.9	—	—	39.1
7	Fluoranthene	9.22	22.2	2.48	19.8	22.9	—	0.60	1.73	0.40	10.4	1.75	1.37	13.8	7.58	14.0	49.7	99.0	2.11	1.38	44.5
8	Pyrene	6.07	35.0	9.45	34.1	20.5	9.46	77.3	113	3.11	178	112	20.3	358	34.2	55.7	121	98.4	3.21	1.42	67.2
9	Benzo[a]anthracene	0.43	6.26	6.28	8.32	1.69	1.67	1.03	1.22	1.13	1.64	1.45	2.50	4.78	4.46	27.7	9.04	51.9	1.19	0.77	2.06
10	Chrysene	0.24	6.18	1.16	8.42	1.04	—	—	—	—	—	0.58	—	0.70	7.22	0.59	42.8	262	1.11	—	58.5
11	Benzo[b]fluoranthene	0.48	11.5	14.8	4.92	1.55	—	—	—	—	13.6	—	—	—	23.3	0.21	37.4	128	1.31	1.96	28.2
12	Benzo[k]fluoranthene	0.16	14.7	10.9	18.3	4.77	—	—	—	—	3.86	—	—	0.01	3.49	8.33	10.2	10.6	—	0.53	0.54
13	Benzo[a]pyrene	— [b]	3.79	1.99	2.12	1.04	0.36	0.47	0.59	0.35		0.73	0.33	1.11	1.49	0.57	1.74	14.4	—	0.34	4.68
14	Dibenzo[a,h]anthracene	0.55	4.50	8.50	3.05	1.94	2.32	1.10	0.81	1.23	9.60	1.81	2.34	1.97	7.38	3.56	14.4	75.7	2.22	0.90	9.94
15	Indeno[1,2,3-c,d]pyrene	0.75	49.8	83.4	60.9	41.1	3.05	1.68	0.48	6.28	2.35	11.7	1.40	27.3	116	54.8	154	184	2.64	0.42	184
16	Benzo[g,h,i]perylene	—	5.39	14.1	4.07	0.81	—	—	—	—	4.38	—	—	—	2.15	0.64	2.87	47.1	—	0.36	16.9
	∑PAH	29.5	216	251	202	116	29.9	85.2	125	45.3	234	145	35.8	497	241	231	543	1228	59.5	15.5	528
	BaP_{eq} ($mg{\cdot}kg^{-1}$)	0.74	12.8	16.3	9.59	4.31	2.77	1.68	1.54	1.71	12.1	2.87	2.76	4.01	13.8	6.3	27.7	139	2.55	1.55	27.5

[a] The reporting limit for individual compounds in $PM_{2.5}$ was 0.23 $mg{\cdot}kg^{-3}$.

[b] —: Not detected.

Continuous **Table S2** $PM_{2.5}$ mass ($\mu g \cdot m^{-3}$) and its polycyclic aromatic hydrocarbons (PAHs) concentrations ($mg \cdot kg^{-1}$) during winter in 2020 (n = 39)

	Sample No.	21	22	23	24	25	26	27	28	29	30	31	32	33	34	35	36	37	38	39
	Date	11.26	11.27	11.28	11.29	11.3	12.1	12.3	12.4	12.5	12.6	12.7	12.1	12.11	12.13	12.14	12.15	12.16	12.17	12.18
	$PM_{2.5}$	88	106	63	67	70	118	57	59	45	111	47	75	61	21	59	44	52	60	69
1	Naphthalene	24.6 [a]	98.4	18.0	252	46.5	39.3	132	114	134	582	469.	149	47.2	66.5	—	96.2	124	4.73	18.6
2	Acenaphthylene	2.21	11.7	4.52	241	10.5	7.73	133	11.9	3.13	11.4	7.68	11.3	9.98	—	—	11.2	39.9	4.13	0.92
3	Fluorene	— [b]	4.55	—	6.22	1.60	2.41	—	0.55	6.63	5.26	10.6	11.9	5.70	7.39	—	—	13.8	13.6	2.08
4	Acenaphthene	11.3	—	4.79	0.04	23.4	12.4	16.2	71.4	130	—	56.7	80.8	24.9	64.6	0.51	82.7	72.7	82.5	85.0
5	Phenanthrene	—	0.70	48.9	10.7	—	18.3	—	32.5	21.7	13.1	15.3	67.2	14.7	13.8	—	15.2	73.3	32.2	—
6	Anthracene	42.4	4.46	26.5	39.0	99.2	77.1	62.9	274	264	180	132	214	73.9	162	0.72	187	192	193	137
7	Fluoranthene	55.8	1.12	30.8	50.1	219	115	108	340	193	191	170	176	50.3	160	3.45	191	197	148	129
8	Pyrene	126	15.7	52.7	91.9	51.5	107	87.9	156	9.61	88.5	468	128	69.3	181	7.54	59.6	194	179	16.2
9	Benzo[a]anthracene	3.04	0.72	3.18	20.3	22.7	5.97	9.25	46.2	29.5	122.	9.13	16.9	6.29	7.87	1.21	3.87	14.1	15.3	7.61
10	Chrysene	17.9	1.44	21.1	8.21	120	40.1	58.5	188	186	8.18	57.6	76.4	37.1	62.9	1.79	0.64	82.8	66.4	7.20
11	Benzo[b]fluoranthene	54.7	1.32	28.1	46.6	132	18.2	17.7	169	222	9.74	69.2	181	71.2	62.2	0.70	150	92.1	52.2	27.9
12	Benzo[k]fluoranthene	19.5	7.06	8.92	—	54.2	6.53	5.56	63.5	93.3	127	44.3	64.5	24.2	40.2	—	52.7	55.9	41.6	17.8
13	Benzo[a]pyrene	4.21	5.03	0.79	16.6	14.2	3.99	2.17	15.1	16.0	49.1	4.43	6.34	4.28	4.14	0.24	7.31	9.83	4.84	7.38
14	Dibenzo[a,h]anthracene	23.4	5.05	12.9	15.2	63.8	19.7	30.6	47.1	8.79	4.51	15.7	18.5	17.9	13.6	0.54	6.32	8.56	11.2	22.1
15	Indeno[1,2,3-c,d]pyrene	51.5	39.9	6.61	220	46.5	259.4	45.6	475	301	298	315	9.46	188	312	14.9	245.0	939	440	41.5
16	Benzo[g,h,i]perylene	2.11	—	3.24	8.75	10.3	11.7	—	17.0	35.3	0.82	17.3	44.8	20.1	33.1	—	40.2	39.1	35.0	28.7
	∑PAH	439	197	271	1027	915	745	709	2021	1654	1691	1862	1256	665	1191	31.6	1149	2148	1324	549
	BaP_{eq} ($mg \cdot kg^{-1}$)	38.2	11.6	20.3	41.6	111.7	35.1	42.6	115	84.9	75.1	44.7	64.5	40.3	42.9	1.2	42.8	57.5	42.5	39.8

[a] The reporting limit for individual compounds in $PM_{2.5}$ was 0.23 $mg \cdot kg^{-1}$.

[b] —: Not detected.
